# Data-Driven Vaccine Clinical Trial Design Features and Associated Progress Patterns: An Analysis of 1618 Clinical Trials from 2012 to 2022

**DOI:** 10.3390/vaccines14060489

**Published:** 2026-05-30

**Authors:** Siyang Chan, Dachuang Zhou, Di Zhang, Yuting Xia, Wenxi Tang

**Affiliations:** 1 Center for Pharmacoeconomics and Outcomes Research, China Pharmaceutical University, Nanjing 211198, China; 3224041316@stu.cpu.edu.cn (S.C.); 3122044202@stu.cpu.edu.cn (D.Z.); 3224041315@stu.cpu.edu.cn (D.Z.); xyt_shama@stu.cpu.edu.cn (Y.X.); 2 Department of Public Affairs Management, School of International Pharmaceutical Business, China Pharmaceutical University, Nanjing 211198, China

**Keywords:** vaccine trials, clinical development, trial success determinants, machine learning, evidence-based design, R&D efficiency

## Abstract

Background/Objectives: Vaccine clinical trials face high costs, long timelines, and variable progression rates, yet systematic evidence linking trial design features to progression outcomes remains limited. This study aimed to identify trial design features associated with vaccine trial progression and to explore robust design configurations using machine learning approaches. Methods: We analyzed 1618 vaccine trials registered from 2012 to 2022. Progression was defined as phase advancement (phase I/II) or regulatory authorization (phase III). Logistic regression assessed associations with progression. Random forest classifiers with cross-validation were used to estimate predicted progression probabilities based on combinations of design features. Monte Carlo simulations compared model-identified robust configurations with randomly generated configurations. Results: Among 1618 trials, 579 achieved phase progressions, corresponding to an overall observed progression rate of 35.8%. Larger sample size, preventive vaccine purpose, COVID-19 indication, and enrollment across all age groups were consistently associated with higher observed odds of progression in both univariable and multivariable logistic regression analyses. In machine learning analyses, the pooled mean predicted progression probability of model-identified robust configurations was 48.93%, compared with 39.44% for historically observed design configurations, corresponding to a relative increase of 24.1%. Simulations further showed a lower projected cumulative development duration (106.87 vs. 128.25 months; −16.7%) and reduced projected cost (USD 100.67M vs. USD 108.33M; −7.1%) for robust configurations compared with historical strategies. Conclusions: This study provides a data-driven framework for characterizing historical vaccine trial design patterns. By integrating machine learning with observational registry data, it supports hypothesis generation and descriptive benchmarking of design features that may inform the design of future prospective or causal investigations.

## 1. Introduction

Vaccines are indispensable for preventing disease, averting deaths, and improving global health. The World Health Organization (WHO) estimates that vaccination has averted an average of 4 million deaths annually in recent years [1]. Beyond reducing disease burden, rapid vaccine development and deployment played a critical role in restoring socioeconomic activity during the COVID-19 pandemic [2].

Yet many infectious diseases still lack effective vaccine protection, and the threat posed by emerging pathogens persists. Vaccine research and development therefore remain essential to safeguarding population health [3]. In public health emergencies, rapid vaccine development is especially critical, clinical trials often account for a substantial proportion of the pre-licensure timeline, with estimates exceeding 50% in some analyses [4,5]. Improving the understanding of clinical trial design features may contribute to characterizing current development patterns. Notably, many setbacks in vaccine development may be related not only to biological or technical hurdles, but also to the absence of robust reference metrics and quantitative frameworks. This gap may contribute to trial designs that are effectively ad hoc, leading to high costs, prolonged timelines, and unsustainable burdens for sponsors [6,7].

Data-driven reference metrics and quantitative frameworks may be useful for characterizing patterns in vaccine trial design and progression outcomes. Although the number of vaccine clinical trials has generally increased year-on-year, progression rates have not improved markedly [8], highlighting substantial heterogeneity in clinical development pathways across diseases, phases, and development settings. In broader clinical development research, data-driven analytical approaches have been used to examine historical trial characteristics, progression patterns, and development variability across therapeutic areas [9,10,11]. However, such approaches remain less commonly applied in vaccine trial research, which has focused primarily on immunogenicity prediction, antigen selection, and safety signal detection [12]. Comparatively less attention has been given to large-scale characterization of trial feature structures and empirical progression patterns across heterogeneous vaccine development settings.

Existing studies are also frequently constrained by limited scope (e.g., single vaccine platforms or narrow therapeutic areas), relatively small datasets, or analytical approaches focused mainly on isolated associations rather than multidimensional feature structure [5,8,13,14]. As a result, current evidence remains fragmented and provides limited insight into how observable trial characteristics cluster and vary across historical vaccine development programs.

To address these gaps, we assembled a near-comprehensive dataset of vaccine clinical trials conducted over the past decade. This study provides three descriptive analytical components: (1) a structured characterization of trial design features and observed progression outcomes; (2) identification of recurrent design configurations within disease- and phase-specific subsets, using random forests as an exploratory pattern-recognition tool (not as a predictive engine); and (3) exploratory scenario-based comparisons of projected time and cost distributions under different historical configuration distributions. Conventional regression analyses were used to examine statistical associations between observable trial characteristics and progression outcomes, whereas machine learning methods were applied to summarize recurring high-dimensional configuration patterns within the observed dataset. The resulting analyses were retrospective and exploratory in nature and were intended to support structured characterization of historical trial design patterns rather than prospective optimization or prescriptive trial design guidance. Collectively, this work contributes to a data-enabled framework for examining variability and recurrent configuration structures in vaccine clinical trial development.

## 2. Methods

### 2.1. Study Design

We assembled a comprehensive registry-based dataset of vaccine clinical trials conducted over the past decade. Trial progression outcomes were first descriptively summarized across major trial design features, followed by univariable and multivariable analyses to examine statistical associations between trial characteristics and progression outcomes. Multivariable models were specified based on clinical and methodological relevance together with data availability considerations.

Machine learning analyses were conducted within disease- and phase-specific subsets to identify important trial design features associated with observed progression outcomes. A random forest model was used to estimate variable importance and to summarize key feature patterns related to higher progression probabilities.

All analyses were retrospective and exploratory. Robust-oriented configurations were then identified exploratorily based on consistency of important features and relatively stable predicted progression probabilities across disease and phase subsets, supported by sensitivity analyses using different minimum sample size thresholds.

Exploratory scenario-based comparisons were performed to examine differences in projected development time and cost between robust-oriented configurations and historical configuration patterns.

### 2.2. Data Sources and Extraction

**Figure 1:**
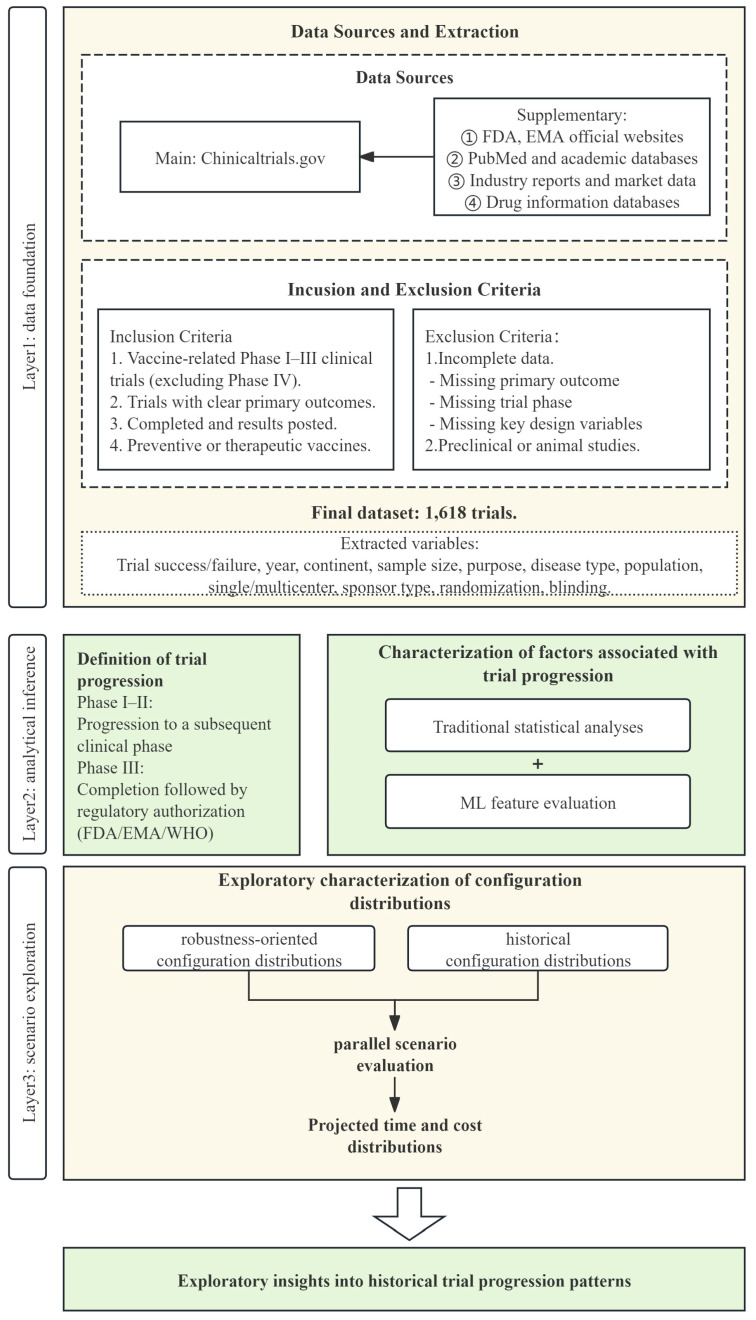
Study design.

As shown in Figure 1, we identified vaccine clinical trials registered on ClinicalTrials.gov between 1 January 2012 and 31 December 2022 through a structured registry search using the term “vaccine” [15]. Registry-derived data were supplemented with publicly available regulatory and development-status sources, including FDA records and related databases [16,17,18,19]. Variables were extracted from predefined registry fields, and records with missing or ambiguous key variables required for analysis were excluded according to prespecified criteria. After exclusion of incomplete records and non-human studies, 1618 clinical trials were included in the final dataset. Detailed inclusion and exclusion procedures are provided in the Supplementary file (pp. 3–4). Because the dataset was based on registry-reported information, reporting delays and incomplete updates may remain.

Operational definitions of trial progression outcomes were prespecified and interpreted as proxy measures of observable development progression rather than definitive indicators of clinical or regulatory success. For phase I–II trials, progression was defined as documented advancement to a subsequent trial phase. Trials without evidence of progression were classified as non-progressed based on available registry records, although absence of follow-up may reflect delayed reporting or incomplete updates. For phase III trials, progression was defined as trial completion with subsequent regulatory authorization where identifiable from public records. Follow-up was extended through 1 June 2025 to reduce potential right-censoring.

To improve interpretability and reduce overcategorization, feature variables were harmonized using predefined grouping rules based on registry fields and methodological considerations [20,21]. Feature definitions and grouping methods were prespecified based on trial registry fields and relevant methodological considerations. Continuous variables (e.g., sample size) were categorized into quantile-based groups to ensure balanced sample sizes and improve interpretability, while categorical variables (e.g., trial phase, vaccine purpose, disease type, age group, study design, and funding source) were harmonized using standard classifications from ClinicalTrials.gov and established frameworks (e.g., ICD-10). Categories with small sample sizes were combined where appropriate to improve stability and interpretability. Detailed definitions and grouping procedures are provided in the Supplementary file (pp. 5–12). Potential collinearity among variables was considered conceptually, and results were interpreted with caution where structural overlap may exist. We summarized trial characteristics and progression outcomes across 13 feature domains, including year, continent, and sample size.

### 2.3. Identification of Factors Associated with Trial Progress

Univariable logistic regression analyses were initially performed to examine associations between individual trial design characteristics and progression outcomes across major feature domains, including sample size, vaccine purpose, disease type, age group, study center structure, funding source, randomization, control, and blinding [22]. Crude odds ratios (ORs) with 95% confidence intervals (CIs) were estimated for each variable.

Candidate variables for multivariable modeling were determined based on a combination of observed associations, methodological relevance, and data availability considerations. These variables were subsequently included in multivariable logistic regression models to estimate adjusted associations within the observed dataset. Adjusted ORs with 95% CIs were reported. For categorical variables, overall *p* values were calculated using likelihood ratio tests (LRTs) to assess the overall association between each variable and progression outcomes. Reference categories were defined using common or representative groups to facilitate interpretation. Results are presented as odds ratios (ORs) with 95% confidence intervals (CIs), where ORs greater than 1 indicate positive associations with progression outcomes [23].

All regression analyses were exploratory and descriptive in nature and were intended to characterize statistical associations within the historical dataset rather than to establish causation or independent predictive effects.

### 2.4. Machine Learning-Based Analysis of Trial Design Features and Robustness Assessment

Machine learning methods were applied to characterize recurrent patterns in trial feature configurations and to evaluate the stability of progression behavior across disease- and phase-specific subsets within the historical dataset. The framework aimed to support retrospective pattern characterization under the observed data distribution, not to generate prescriptive recommendations or prospective predictions [24,25].

A Random Forest classifier was chosen as the primary model due to its flexibility with mixed-type variables and its ability to model complex non-parametric relationships without explicit functional assumptions [26]. The model consists of an ensemble of 1000 decision trees grown using bootstrap sampling, with final predictions averaged across all trees to improve the stability of cross-validated outputs [27].

To mitigate overfitting and generate cross-validated estimates, we applied stratified 5-fold cross-validation [28]. In each fold, the model was trained on a subset of the data and evaluated on the held-out fold, producing out-of-fold (OOF) estimates for all observations. These OOF estimates were then used for configuration-level aggregation and robustness-oriented characterization analyses.

Variable importance was quantified using the mean decrease in Gini impurity from the fitted Random Forest model. To reduce dimensionality and avoid excessive fragmentation of configuration groups, only the five most influential variables were retained for subsequent aggregation and robustness analyses. Trials were grouped by identical combinations of these predefined strategy variables. For each configuration group, we calculated the observed progression proportion and Wilson-type 95% confidence intervals to quantify uncertainty [29]. Additionally, empirical Bayes shrinkage with a Beta(1,1) prior was applied to regularize observed proportions in sparse groups.

Configuration groups were ordered using a robustness-oriented framework primarily based on the lower bound of the Wilson type 95% confidence interval, together with empirical progression proportions and agreement between empirical and cross-validated model-derived estimates.

In the primary analysis, only groups containing at least 10 observations were summarized at the configuration level to reduce instability from sparse feature combinations. This restriction was applied only during post hoc aggregation and did not affect model fitting or OOF estimation.

Configuration groups that simultaneously showed higher empirical progression proportions, higher lower-confidence-bound estimates, and closer agreement between empirical and model-derived estimates were interpreted as robustness-oriented configuration patterns.

Sensitivity analyses were performed using alternative minimum sample-size thresholds of n = 5 and n = 15, in addition to the primary threshold of n = 10 (Supplementary file pp. 14–15). Consistency of high-ranking configuration groups across thresholds was examined to evaluate the robustness of observed patterns under varying sparsity conditions.

### 2.5. Simulation-Based Characterization of Configuration-Level Modeled Distributions

Exploratory simulation analyses were conducted to examine how different historical configuration distributions were associated with projected trial counts, cumulative development time, and cumulative development cost under standardized hypothetical assumptions.

Two configuration-distribution scenarios were evaluated. The first was based on historically observed configuration frequencies within each disease–phase subgroup. The second used robustness-oriented configuration distributions identified through the machine learning aggregation framework. The comparison was intended to explore whether concentration toward configurations showing greater empirical consistency would yield different modeled resource use distributions under simplified assumptions.

Progression probabilities were modeled using Beta distributions parameterized from observed progression proportions and corresponding sample sizes. Trial progression was then simulated iteratively until progression to the subsequent development stage occurred, with the number of required trials generated using a geometric process framework.

Phase-specific development time and cost parameters were derived from previously published estimates of vaccine and oncology clinical development studies [7,30,31,32]. To improve comparability across studies conducted in different periods, monetary values were inflation-adjusted to 2025 US dollars using Consumer Price Index data from the US Bureau of Labor Statistics [33]. Time and cost parameters were sampled from log-normal distributions to incorporate uncertainty in the simulation framework.

Simulated phase I–III results were subsequently aggregated to estimate projected cumulative trial counts, development time, and development cost across repeated iterations. The results are presented as summary distributions, including means, medians, and empirical uncertainty intervals.

The simulation framework generated standardized scenario-based projections of trial counts, development time, and development cost across historical and robustness-oriented configuration distributions.

All analyses were conducted in R (version 4.3.2).

## 3. Results

### 3.1. Overall Progression Rates of Vaccine Clinical Trials

Among 1618 vaccine clinical trials included in the analysis, 579 demonstrated documented progression to the subsequent development stage, corresponding to an overall observed progression rate of 35.80%, broadly consistent with previous reports [8]. From 2012 to 2021, the annual number of registered vaccine trials generally increased, followed by a decline in 2022. Observed progression rates decreased between 2012 and 2017 and subsequently increased during 2018–2021, reaching the highest level in 2021 Supplementary file (p. 2).

Regionally, the Americas contributed the largest number of trials. Oceania showed the highest observed progression rate despite having the fewest trials, whereas Africa showed the lowest observed progression rate. These differences should be interpreted cautiously, as regional variation in trial composition, disease focus, and development context may contribute to the observed patterns. Detailed results across trial characteristics are presented in Table 1.

### 3.2. Factors Associated with Vaccine Trial Progression

In univariable logistic regression analyses, larger sample size, preventive vaccine purpose, COVID-19 indication, enrollment across all age groups, industry sponsorship, multi-arm trial structures, and the presence of blinding were associated with higher observed odds of progression within the study dataset. Randomization scheme was not significantly associated with progression outcomes.

In multivariable logistic regression analyses, larger sample size, preventive vaccine purpose, COVID-19 indication, and enrollment across all age groups remained associated with higher observed odds of progression. In contrast, associations for study center structure, intervention model, and blinding were attenuated after adjustment and were no longer statistically significant. Funding source remained associated with progression outcomes overall, although estimates for network/collaborative alliance-sponsored trials were imprecise because of the small sample size in this subgroup.

These findings should be interpreted as descriptive associations within the historical registry dataset rather than evidence of causal or independently predictive relationships. Full regression results are presented in Table 1.

### 3.3. Machine Learning-Based Analysis of Trial Design Features and Model-Based Comparisons

In the machine learning models, the variables with the top 5 highest importance included phase, disease type, age group, funding source, and purpose of vaccine (Supplementary file p. 13). After applying the minimum sample size threshold (n ≥ 10) to the original dataset, a total of 42 unique feature combinations were retained for strategy-level analysis across all disease phase subsets. Examples of the highest-ranked trial design configurations (by disease and phase) identified within the modeling framework are shown in Table 2.

**Figure 2:**
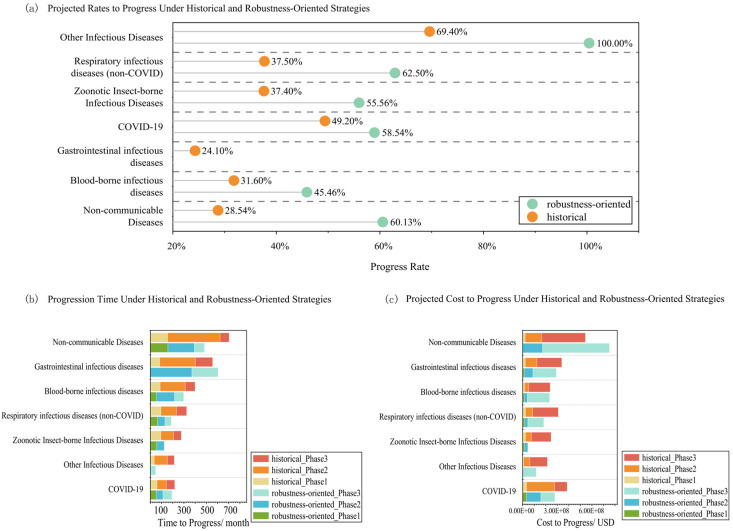
Distribution of configuration-level modeled progression estimates under alternative aggregation approaches. (**a**) Projected rates to progress under historical and robustness-oriented strategies; (**b**) Progression time under historical and robustness-oriented strategies; (**c**) Projected cost to progress under historical and robustness-oriented strategies.

As shown in Figure 2, configurations identified by the model as robustness-oriented were associated with relatively higher model-derived progression probabilities compared with the overall distribution of historical configurations. The pooled mean predicted progression probability was 48.93% for robust-oriented configurations versus 39.44% in the historical distribution; however, the two distributions showed substantial overlap.

Under standardized assumptions for per-trial duration and cost, scenario-based analyses suggested differences in projected time and cost distributions between the two groups. The weighted mean projected duration was 106.87 months for robust-oriented configurations compared with 128.25 months for historical configurations. The corresponding weighted mean projected cost was USD 100.67 million versus USD 108.33 million, respectively.

Monte Carlo simulation scatter plots comparing model-based and historical strategy distributions are presented in the Supplementary file (p. 16). Across the simulated scenarios, the model-based configurations showed a modest tendency toward higher progression probabilities together with lower projected cumulative time and cost, although substantial overlap between the two distributions remained.

## 4. Discussion

Using 1618 global vaccine clinical trials, this study provides a structured characterization of historical associations between observable trial design features and progression outcomes within registry-based data. Larger sample size, preventive vaccine purpose, enrollment across all age groups, and COVID-19 indication remained associated with higher observed progression odds after multivariable adjustment, whereas several operational characteristics, including multicenter structure and blinding, showed attenuated associations after adjustment. Some observed associations likely reflect broader scientific, operational, and regulatory contexts surrounding vaccine development rather than isolated effects of individual design characteristics. For example, differences in blinding strategy, study structure, and funding source may partially capture variation in trial phase, development urgency, endpoint selection, or sponsor resources. Accordingly, these findings should be interpreted as descriptive patterns embedded within historical development environments rather than evidence of causal relationships or prescriptive trial design advantages. Temporal trends indicated that vaccine trial activity increased steadily between 2012 and 2021, coinciding with expansion of global vaccine development efforts during the COVID-19 period [31,34,35]. However, COVID-19-related trials did not uniformly exhibit the highest progression proportions across all disease categories [15]. This further suggests that progression outcomes are shaped by complex interactions among scientific, epidemiological, regulatory, and operational factors that cannot be fully disentangled within registry-based observational data.

The machine learning framework summarized recurrent high-dimensional feature combinations observed across historical vaccine trials and highlighted disease–phase-specific configuration patterns associated with comparatively stable progression behavior within the dataset. By focusing on the five most influential variables identified by the model, the analysis highlighted configuration groups showing higher empirical progression proportions together with greater consistency between empirical and model-derived estimates. Robust-oriented configurations within this framework were associated with higher model-estimated progression probabilities and modestly lower simulated cumulative time and cost under standardized assumptions compared with historical strategy distributions. However, substantial overlap remained between the simulated distributions, indicating that the observed differences were incremental and scenario-dependent. Collectively, descriptive configuration assessment may provide a useful exploratory framework for characterizing historical progression patterns across heterogeneous vaccine development settings.

This study has limitations. First, reliance on ClinicalTrials.gov and supplementary public databases may introduce selection bias, reporting delays, and incomplete outcome capture [21]. Trials lacking documented progression were operationally classified as non-progression, which may underestimate true progression rates when follow-up information was delayed or unavailable [8,36]. Although follow-up through 1 June 2025 was used to reduce right-censoring, some outcomes likely remained unobserved. Second, as an observational registry-based study, the analysis is inherently vulnerable to confounding and omitted variable bias. Important determinants of vaccine development outcomes—including immunogenicity, prior preclinical evidence, sponsor expertise, manufacturing feasibility, regulatory interactions, and epidemic dynamics—were not comprehensively captured in the available data. Consequently, observed associations may largely reflect broader development environments rather than direct relationships between individual trial features and progression outcomes. Third, although Random Forest modeling and internal cross-validation were used to summarize configuration patterns, the ranking framework depended on predefined aggregation procedures, weighting choices, and sparsity thresholds. Alternative specifications could yield different configuration rankings and simulation outputs. Future work should focus on external validation across independent datasets, alternative model specification strategies, and more formal causal or decision-analytic frameworks to assess whether any observed configuration patterns remain stable under broader development conditions.

## 5. Conclusions

This study describes progression patterns in vaccine clinical trials using a large historical registry dataset. Sample size, vaccine purpose, target population, and funding source showed variable associations with trial progression across disease areas and development phases.

A machine learning approach was used to summarize common combinations of trial design features observed in historical data and to identify groups of configurations that showed relatively consistent progression outcomes within the study dataset. Scenario analyses showed that these robust-oriented configurations were associated with small differences in projected development time and cost compared with historical trial design patterns.

Overall, this study provides a descriptive overview of how vaccine trial design features relate to progression outcomes in historical data. Further validation using external datasets and prospective evaluation is needed before any broader application.

## Figures and Tables

**Table 1:** Univariable Analysis and Multivariable Analysis of Factors Associated with Vaccine Trial Progression.

Category	Total n (%)	Progressed n (%)	Crude OR *	Univariable *p*	Adjusted OR (95% CI)	Multivariable *p*
**Sample size**						
0–49	423 (26.14%)	86 (20.30%)	-	*p* < 0.001	-	*p* = 0.002
50 –99	324 (19.99%)	89 (27.40%)	1.48 (1.02–2.14)		1.01 (0.62–1.62)	
100–299	274 (16.93%)	107 (39.20%)	2.53 (1.83–3.51)		1.69 (1.08–2.66)	
300–999	356 (21.99%)	156 (43.80%)	3.06 (2.23–4.21)		1.93 (1.20–3.13)	
1000+	241 (14.28%)	127 (52.60%)	4.34 (3.11–6.09)		2.56 (1.53–4.33)	
**Purpose of vaccine**						
Preventive	1221 (75.46%)	518 (42.40%)	-	*p* < 0.001	-	*p* < 0.001
Therapeutic	320 (19.77%)	46 (14.40%)	0.23 (0.16–0.31)		0.40 (0.22–0.71)	
Other	77 (4.76%)	15 (19.50%)	0.33 (0.18–0.57)		0.39 (0.17–0.80)	
**Disease type**						
COVID-19	171 (10.93%)	84 (49.20%)	-	*p* < 0.001	-	*p* < 0.001
Other infectious diseases	307 (19.03%)	213 (69.40%)	2.34 (1.32–4.28)		2.54 (1.24–5.43)	
Blood-borne infectious diseases	87 (5.38%)	27 (31.60%)	0.48 (0.32–0.70)		0.56 (0.32–0.97)	
Zoonotic/vector-borne infectious diseases	254 (15.69%)	95 (37.40%)	0.62 (0.43–0.88)		0.67 (0.42–1.06)	
Respiratory infectious diseases (non-COVID)	62 (3.83%)	23 (37.50%)	0.62 (0.47–0.82)		0.51 (0.34–0.74)	
Gastrointestinal infectious diseases	523 (32.33%)	126 (24.10%)	0.33 (0.19–0.56)		0.29 (0.15–0.57)	
Non-communicable diseases	214 (13.23%)	29 (13.40%)	0.16 (0.10–0.24)		0.86 (0.39–1.90)	
**Age group**						
All ages	76 (4.70%)	43 (56.60%)	-	*p* < 0.001	-	*p* < 0.001
18–64	498 (30.78%)	176 (35.30%)	0.42 (0.26–0.68)		0.48 (0.24–0.94)	
0–17	235 (14.52%)	81 (34.50%)	0.40 (0.24–0.68)		0.27 (0.13–0.55)	
65+	40 (2.47%)	13 (31.60%)	0.94 (0.43–2.04)		0.59 (0.23–1.51)	
0–64	115 (7.11%)	60 (52.20%)	0.84 (0.47–1.50)		0.66 (0.30–1.41)	
18+	654 (40.42%)	197 (30.10%)	0.33 (0.20–0.54)		0.28 (0.14–0.54)	
**Single-/multi-center**						
Multicenter	1041 (64.35%)	332 (31.90%)	-	*p* = 0.015	-	*p* = 0.864
Single center	577 (35.66%)	219 (37.90%)	1.31 (1.05–1.62)		1.03 (0.76–1.38)	
**Funding source**						
Industry	1019 (63.01%)	449 (44.10%)	-	*p* < 0.001	-	*p* < 0.001
Network/collaborative alliance	6 (0.37%)	3 (50.00%)	0.25 (0.01–1.58)		0.36 (0.02–2.63)	
Government/public sector	227 (1.67%)	56 (24.70%)	0.47 (0.34–0.64)		0.50 (0.33–0.73)	
Academic institutions/non-profit organizations	366 (22.62%)	68 (18.60%)	0.29 (0.22–0.39)		0.36 (0.24–0.55)	
**Randomization**						
Randomized design	1202 (74.29%)	453 (37.70%)	-	*p* = 0.587	-	*p* = 0.52
Non-randomized design	416 (25.71%)	151 (36.40%)	0.94 (0.69–1.29)		0.94 (0.69–1.29)	
**Control**						
Single-group design	239 (14.77%)	61 (25.51%)	-	*p* < 0.001	-	*p* = 0.18
Crossover design	10 (0.62%)	5 (50.00%)	2.98 (0.80–11.07)		2.06 (0.32–13.87)	
Factorial design	9 (0.56%)	4 (44.40%)	2.39 (0.57–9.30)		2.84 (0.44–17.63)	
Parallel design	1185 (73.18%)	463 (39.10%)	1.91 (1.40–2.64)		1.78 (0.65–5.35)	
Sequential design	173 (10.69%)	45 (26.00%)	1.05 (0.67–1.64)		1.08 (0.37–3.42)	
**Blinding**						
With blinding	987 (60.99%)	379 (38.40%)	-	*p* = 0.006	-	*p* = 0.08
Without blinding	631 (39.00%)	200 (31.70%)	0.74 (0.60–0.92)		0.73 (0.51–1.03)	
**Type of blinding**						
No blinding	631(39.00%)	200 (31.70%)	-	*p* = 0.048	-	*p* = 0.39
Single-blind	67 (45.14%)	26 (38.80%)	1.37 (0.80–2.28)		0.98 (0.51–1.87)	
Double-blind	307 (19.03%)	112 (36.40%)	1.23 (0.92–1.65)		0.62 (0.40–0.97)	
Triple-blind	215 (13.29%)	91 (42.30%)	1.58 (1.15–2.17)		0.72 (0.45–1.15)	
Quadruple-blind	400 (24.72%)	151 (37.80%)	1.31 (1.00–1.70)		0.59 (0.39–0.91)	

* Crude and adjusted odds ratios (ORs) with 95% confidence intervals (CIs) were estimated using univariable and multivariable logistic regression models, respectively. For categorical variables, overall *p* values were calculated using likelihood ratio tests (LRTs).

**Table 2:** Examples of top-ranked trial design configurations by disease and phase within the modeling framework.

Disease Type	Phase	Age Group	Funding Source	Purpose of Vaccine	Progression Rate	Wilson 95% Confidence Interval *
COVID-19	Phase 1	18–64	Industry	Prevention	58.33%	38.83%
COVID-19	Phase 2	18–64	Industry	Prevention	63.64%	35.38%
COVID-19	Phase 3	18+	Industry	Prevention	57.45%	43.28%
Blood-borne infectious diseases	Phase 1	18–64	Industry	Prevention	60.00%	31.26%
Blood-borne infectious diseases	Phase 2	18–64	Industry	Prevention	23.08%	9.75%
Blood-borne infectious diseases	Phase 3	0–64	Industry	Prevention	60.00%	31.27%
Gastrointestinal infectious diseases	Phase 2	18–64	Industry	Prevention	10.00%	1.79%
Gastrointestinal infectious diseases	Phase 3	0–18	Industry	Prevention	20.00%	9.29%
Non-communicable disease	Phase 1	18+	Academic institutions/non-profit organizations	Treatment	17.78%	9.29%
Non-communicable disease	Phase 2	18+	Government/public sector	Treatment	11.11%	3.10%
Non-communicable disease	Phase 3	18+	Industry	Treatment	50.00%	36.46%
Other infectious diseases	Phase 3	18+	Industry	Prevention	100.00%	85.13%
Respiratory infectious diseases (non-COVID)	Phase 1	18–64	Industry	Prevention	47.06%	26.16%
Respiratory infectious diseases (non-COVID)	Phase 2	0–64	Industry	Prevention	60.00%	31.27%
Respiratory infectious diseases (non-COVID)	Phase 3	0–64	Industry	Prevention	84.62%	57.76%
Zoonotic/vector-borne disease	Phase 1	18–64	Academic institutions/non-profit organizations	Prevention	53.85%	29.14%
Zoonotic/vector-borne disease	Phase 2	18–64	Industry	Prevention	57.14%	32.59%

* The lower bound of the Wilson 95% confidence interval was used as a conservative estimate of success probability, accounting for sampling variability and reducing the influence of small-sample strategy combinations.

## Data Availability

All data used in this study are publicly available and can be freely accessed and used by other researchers to replicate or extend the findings of this study. All data sources and references are explicitly cited in the manuscript or in the Supplementary Materials.

## Supplementary Materials

The following supporting information can be downloaded at: https://www.mdpi.com/article/10.3390/vaccines14060489/s1, Supplementary file.
