# Supplementary material for: Data-Driven Vaccine Clinical Trial Design Features and Associated Progress Patterns: An Analysis of 1618 Clinical Trials from 2012 to 2022
Source: Vaccines (Basel). 2026 May 30;14(6):489. doi: 10.3390/vaccines14060489 (PMC13307709; doi:10.3390/vaccines14060489)
Supplement: Supplementary file 1 [file vaccines-14-00489-s001.zip › vaccines-4235821-supplementary.pdf]

Supplementary Materials

Contents

Supplementary Materials 1
1. Number and success rate of clinical trials, by year 2
2. Inclusion and exclusion process 3
3. Grouping methods and definition 5
4. Results of variable importance 13
5. Results of sensitivity analysis of sample size cut-offs of n 14
6. Results of probability sensitivity analysis 17
References 18

1. Number and success rate of clinical trials, by year

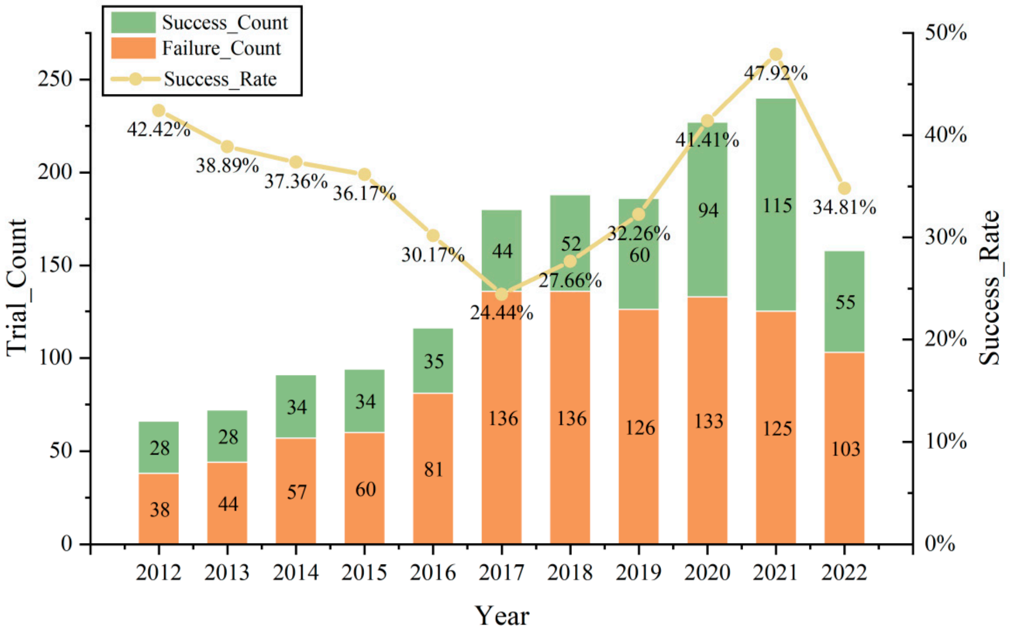

Figure S1. Number and success rate of clinical trials, by year.

2. Inclusion and exclusion process

A comprehensive database search yielded 7,524 initial records. Based on the study period criterion, 5,010 records outside the period 2012–2022 were excluded, leaving 2,514 records within the target time frame. Subsequently, records were screened by study type. Because this study focused on interventional clinical trials, 311 observational studies were excluded, resulting in 2,203 interventional trials retained for further evaluation.

The next step involved screening by trial phase. A total of 584 studies that were not phase I–III were excluded, including 35 early-phase trials, 260 phase IV trials, and 289 trials of unspecified phase. This selection aimed to focus on the core stages of drug development (phases I–III), ensuring scientific rigor and comparability of the analyses.

Phase IV trials were excluded because their research objectives differ substantially from those of phase I–III trials. The latter primarily aim to evaluate the safety and efficacy of vaccines and to support regulatory approval, with success typically defined as progression to the next phase or market authorization. These trials are generally based on clinical data, often employ randomized controlled designs, last from several months to years, and focus on identifying determinants of successful vaccine development.

In contrast, phase IV trials primarily assess the long-term safety and effectiveness of vaccines in real-world settings. Their endpoints focus on detecting rare adverse events or monitoring market performance. They often utilize real-world data and employ observational or cohort designs, with durations extending over many years or even lifelong follow-up. Given these methodological and conceptual differences, only phase I–III trials were included in this analysis.

In total, 1,619 eligible phase I–III clinical trials were ultimately included in the quantitative analysis. This selection process reflects a rigorous screening strategy and systematic study design, ensuring the representativeness and quality control of the analytical dataset.

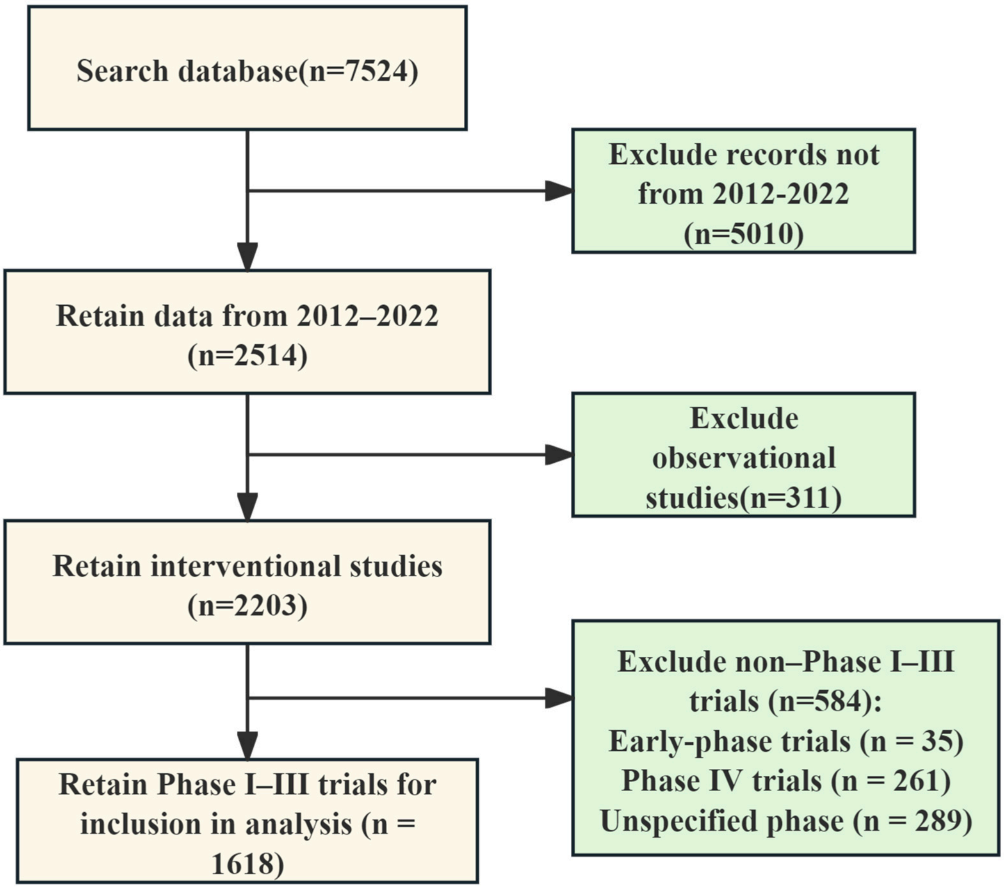

Figure S2. Inclusion and Exclusion Flow Diagram.

3. Grouping methods and definition

Definitions and grouping methods are as follows:

(1) Sample Size

In this study, sample size was defined as a continuous variable representing the number of participants enrolled in each clinical trial. For descriptive analyses, trials were stratified into five categories according to the total number of participants:0–49, 50–99, 100–299, 300–999, and ≥1,000 participants.

The categorization thresholds were defined based on the empirical distribution of sample sizes across the dataset. To avoid having groups with very few or no observations, an unequal-width binning strategy was adopted—using narrower intervals for trials with smaller sample sizes. This approach ensured a more balanced number of trials per group, thereby improving the representativeness and robustness of subsequent statistical analyses.

(2) Trial Phase

The trial phase refers to the stage of a study within the overall drug development process. For the purpose of this analysis, clinical trials were grouped into three categories: Phase I including both phase I and phase I/II trials; Phase II including both phase II and phase II/III trials; Phase III: including phase III trials only.

This classification was based on the trial information provided in the ClinicalTrials.gov database[1]. Phase labels were harmonized to ensure consistency across records, simplifying the categorization and facilitating comparative statistical analysis of success rates and other outcomes across different development stages.

(3) Purpose of vaccine

The primary purpose of each clinical trial was categorized into three groups: prevention, treatment, and other.

Prevention: trials primarily designed to evaluate interventions aimed at disease prevention.

Treatment: trials primarily designed to assess therapeutic interventions for disease management.

Other: trials with purposes other than prevention or treatment, including diagnostic studies, screening studies, health services research, supportive care or ancillary care studies, and basic science investigations.

This classification was based on the “Primary Purpose” field provided in the ClinicalTrials.gov database [1]. Among all included trials, the majority were conducted for prevention (n = 1,220) or treatment (n = 320). Because the number of trials in other categories was relatively small and insufficient for meaningful statistical comparison, all non-prevention trials were consolidated into a single “other” category to enhance the robustness and interpretability of analyses.

(4) Disease Type

Diseases were categorized according to the target conditions that each vaccine aimed to prevent. The classification was based on the Tenth Revision of the International Classification of Diseases (ICD-10)[2], as published by the World Health Organization (WHO) in 2022, with minor simplifications for analytical clarity. Transmission routes for each disease were manually verified through authoritative sources and relevant literature

to ensure the scientific accuracy and internal consistency of classification.

COVID-19: an infectious disease caused by severe acute respiratory syndrome coronavirus 2 (SARS-CoV-2), primarily transmitted via respiratory droplets, contact, and aerosols.

Respiratory diseases: infectious diseases of the respiratory tract other than COVID-19, caused by pathogenic microorganisms (e.g., viruses, bacteria, or mycoplasma) transmitted mainly through droplets, aerosols, or contact. Representative diseases include pneumonia, tuberculosis, and pertussis.

Vector-borne or zoonotic diseases: infections transmitted by animal or arthropod vectors such as mosquitoes, fleas, ticks, or rodents. Typical examples include dengue fever, rabies, yellow fever, and Ebola virus disease.

Blood-borne diseases: infections transmitted through exposure to human body fluids—including blood, semen, vaginal secretions, saliva, breast milk, or cerebrospinal fluid—typically via direct contact or exchange of body fluids. Representative diseases include HIV/AIDS, hepatitis B, and hepatitis C.

Enteric (gastrointestinal) diseases: infections transmitted via the digestive tract, commonly through the fecal–oral route following ingestion of contaminated food, water, or fomites. These diseases primarily affect the gastrointestinal system, with common manifestations such as diarrhea, vomiting, and fever. Examples include cholera, shigellosis, typhoid and paratyphoid fevers, hepatitis A and E, rotavirus or norovirus infection, and amoebic dysentery.

Other infectious diseases: infections with mixed, atypical, or non-exclusive routes of

transmission, such as varicella (chickenpox), monkeypox, and hand, foot, and mouth disease.

Non-infectious diseases: conditions not caused by pathogens but potentially preventable or modifiable through immunological or immune-modulating mechanisms, such as certain cancers, autoimmune disorders, Alzheimer's disease, and allergic diseases.

(5) Age group

Clinical trials were classified according to the age structure of enrolled participants, forming six mutually exclusive categories:

18–64 years: adult participants only.

0–17 years: pediatric participants only.

65+ years: older adult participants only.

18+ years: both adults and older adults included.

0–64 years: both children and adults included.

All ages: participants across all age groups, including children, adults, and older adults.

Age group definitions followed the ClinicalTrials.gov classification: children (0–17 years), adults (18–64 years), and older adults (≥65 years). Because eligibility criteria and study designs varied among trials, some studies included participants spanning more than one age category [1]. Accordingly, each trial was classified based on the combination of age groups represented in its enrolled population.

(6) Single/multicenter

Clinical trials were classified according to the number of participating study sites as

either single-center or multicenter trials.

A single-center trial was defined as a study conducted within a single research institution or clinical center, where all participants were enrolled and managed at one site. These trials generally involve one investigator team, unified data sources, and consistent operational procedures.

A multicenter trial was defined as a study conducted simultaneously at two or more research institutions or clinical centers. Although multicenter trials follow a common study protocol, each site is responsible for conducting the trial independently, typically under the coordination of a lead institution responsible for overall management and data integration.

The single- or multicenter attribute of each trial was determined based on information from the ClinicalTrials.gov database [1], specifically by identifying whether multiple study locations were listed for the trial.

(7) Funding source

Clinical trials were categorized according to the source of primary sponsorship into four groups: industry, academic/non-profit, networks/partnership alliances, and government/public sector.

Industry: sponsors engaged primarily in commercial research and development, including pharmaceutical, biotechnology, and medical device companies.

Academic/Non-profit: universities, research hospitals, academic research centers, and charitable foundations operating on a non-profit basis.

Networks/Partnership Alliances: collaborative networks formed by multiple

institutions, often involving partnerships among academia, industry, and governmental bodies, such as multinational research consortia or public–private partnerships.

Government/Public Sector: national or local governmental funding bodies and public health agencies (e.g., the National Institutes of Health [NIH], Centers for Disease Control and Prevention [CDC], or ministries of health).

Categorization was based on the primary sponsor information reported in the ClinicalTrials.gov database. When multiple sponsoring entities were listed, classification was determined by the organization designated as the lead sponsor.

(8) Trial Design: Randomization

Trials were classified according to their allocation methods as randomized, non-randomized, or not reported (NA).

Randomized trials were those in which participants were assigned to different intervention or control groups using randomization procedures, aimed at minimizing selection bias and strengthening the validity of results.

Non-randomized trials assigned participants based on non-random criteria—such as investigator judgment, participant preference, chronological order, or study center location—rather than by chance.

NA (not reported) indicated that the allocation method was not described in the trial registration record.

Information regarding randomization design was obtained from the ClinicalTrials.gov database [1].

(9) Trial Design: Control

Trials were further classified by study design type as parallel, single-group, crossover, sequential, factorial, or not reported (NA).

Parallel design: participants were assigned to two or more groups that received different interventions concurrently.

Single-group design: all participants received the same intervention, with no control arm.

Crossover design: the same participants received different interventions sequentially, serving as their own controls.

Sequential design: the trial proceeded in stages, with potential modifications or early termination based on interim analyses or pre-specified rules.

Factorial design: two or more interventions or intervention factors were tested simultaneously within the same trial to evaluate individual and interactive effects.

NA (not reported): design type not specified in registration data.

Classification of the intervention model was based on the study information provided in ClinicalTrials.gov [1].

(10) Trial Design: type of blinding

Trials were categorized according to the masking approach as single-blind, double-blind, triple-blind, quadruple-blind, open-label (no blinding), or not reported (NA).

Single-blind: participants were unaware of group assignments, while investigators knew treatment allocation, reducing psychological bias such as placebo or nocebo effects.

Double-blind: both participants and investigators (including clinicians and study personnel) were unaware of group assignments, minimizing bias from both participant

expectations and investigator behavior.

Triple-blind: extended blinding to data analysts or outcome assessors, preventing potential analytical or interpretative bias.

Quadruple-blind: blinding involved participants, investigators, outcome assessors, and trial sponsors or administrators until completion of the study, maximizing protection against bias or external interference.

Open-label (no blinding): both participants and investigators were aware of treatment allocation.

NA (not reported): blinding information not specified in the trial registry.

Blinding classification was determined according to the details provided in the ClinicalTrials.gov database [1].

4. Results of variable importance

Variable	MeanDecreaseAccuracy	MeanDecreaseGini
**Phase**	102.0781	44.1566
**Disease**	91.5397	72.1442
**Funding**	77.0412	35.9416
**Age**	76.7016	61.0804
**Primary_Purpose**	57.1518	21.5312
**Objective**	55.9765	15.7744
**Participants_cat**	44.7286	36.2259
**Blinding**	31.5277	54.2195

5. Results of sensitivity analysis of sample size cut-offs of n

When the sample size cut-off of n was set as n=5, examples of top-ranked trial design configurations by disease and phase within the modeling framework are shown:

Disease type	Phase	Age group	Funding source	Purpose of vaccine	Progression Rate	Wilson 95% confidence interval
COVID-19	Phase 1	18-64	Industry	Prevention	58.33%	38.83%
COVID-19	Phase 2	18-64	Government/public sector	Prevention	83.33%	43.65%
COVID-19	Phase 3	0-64	Industry	Prevention	77.78%	45.26%
Blood-borne infectious diseases	Phase 1	0-64	Industry	Prevention	71.43%	35.89%
Blood-borne infectious diseases	Phase 2	18-64	Industry	Prevention	23.08%	8.18%
Blood-borne infectious diseases	Phase 3	0-64	Industry	Prevention	60.00%	31.27%
Gastrointestinal infectious	Phase 1	18-64	Industry	Prevention	55.56%	26.66%
Gastrointestinal infectious diseases	Phase 2	18-64	Industry	Prevention	10.00%	1.79%
Gastrointestinal infectious diseases	Phase 3	0-18	Industry	Prevention	20.00%	5.67%
Non-communicable disease	Phase 1	18+	Academic institutions/non-profit organizations	Treatment	17.78%	9.29%
Non-communicable disease	Phase 2	18+	Government/public sector	Treatment	11.11%	3.10%
Non-communicable disease	Phase 3	18+	Industry	Treatment	50.00%	36.46%
Other infectious diseases	Phase 3	18+	Industry	Prevention	100.00%	85.13%
Respiratory infectious diseases (non-COVID-19)	Phase 1	18+	Industry	Prevention	47.06%	26.16%

Respiratory infectious diseases (non-COVID-19)	Phase 2	0-64	Industry	Prevention	60.00%	31.27%
Respiratory infectious diseases (non-COVID-19)	Phase 3	0-64	Industry	Prevention	84.62%	57.76%
Zoonotic/vector-borne disease	Phase 1	18-64	Academic institutions/non-profit organizations	Prevention	53.85%	29.14%
Zoonotic/vector-borne disease	Phase 2	18-64	Industry	Prevention	57.14%	32.59%
Zoonotic/vector-borne disease	Phase 3	18+	Industry	Prevention	85.71%	48.69%

When the sample size cut-off of n was set as n=15, examples of top-ranked trial design configurations by disease and phase within the modeling framework are shown:

**Disease type**	**Phase**	**Age group**	**Funding source**	**Purpose of vaccine**	**Progression Rate**	**Wilson 95% confidence interval**
COVID-19	Phase 1	18-64	Industry	Prevention	58.33%	38.83%
COVID-19	Phase 2	18+	Industry	Prevention	40.68%	29.09%
COVID-19	Phase 3	18+	Industry	Prevention	57.45%	43.28%
Non-communicable disease	Phase 1	18+	Academic institutions/non-profit organizations	Treatment	17.78%	9.29%
Non-communicable disease	Phase 2	18+	Government/public sector	Treatment	11.11%	3.10%
Other infectious diseases	Phase 3	18+	Industry	Prevention	100.00%	85.13%
Respiratory infectious diseases (non-COVID-19)	Phase 1	18+	Industry	Prevention	47.06%	26.16%
Respiratory infectious diseases (non-COVID-19)	Phase 2	18-64	Industry	Prevention	32.00%	17.20%
Respiratory infectious	Phase 3	18+	Industry	Prevention	69.23%	53.58%

diseases (non-COVID-19)						
Zoonotic/vector-borne disease	Phase 1	18-64	Industry	Prevention	42.24%	28.67%

6. Results of probability sensitivity analysis

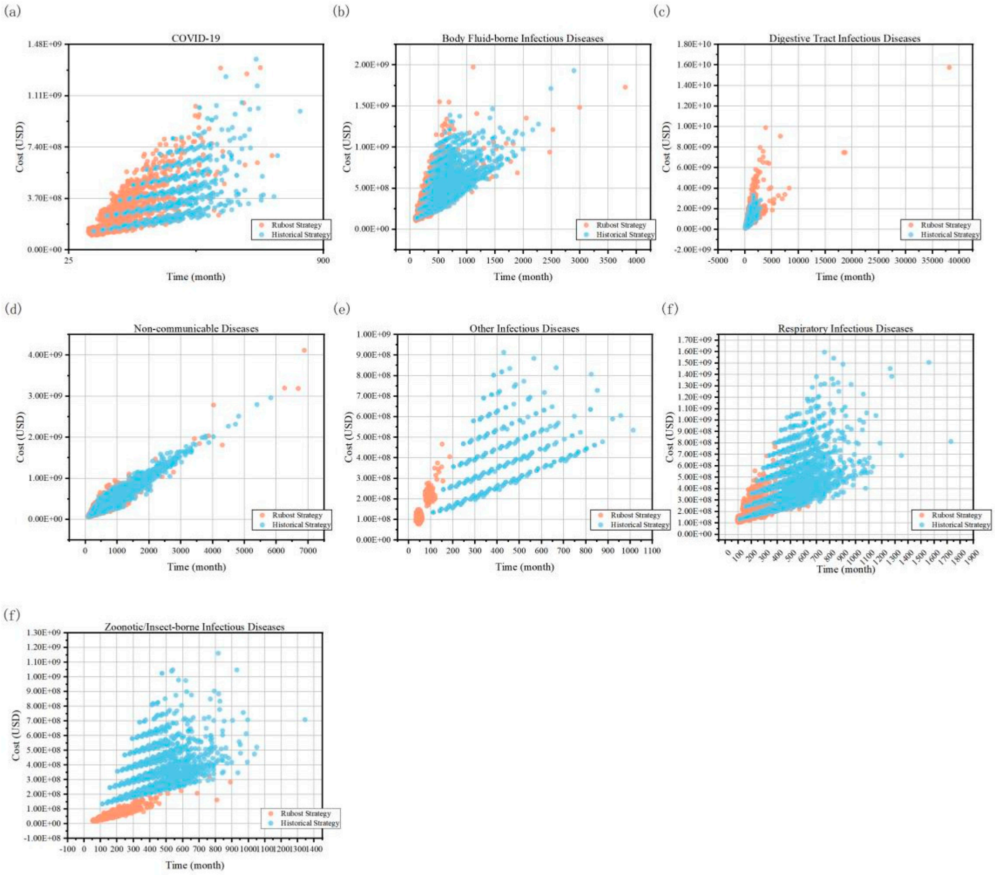

Figure S3. Probabilistic sensitivity analysis (PSA) scatter plots of cost and time by disease category.

References

1. U.S. National Library of Medicine; National Institutes of Health ClinicalTrials.Gov Available online: https://clinicaltrials.gov/ (accessed on 4 September 2025).
2. National Center for Health Statistics; Centers for Disease Control and Prevention *ICD-10, International Statistical Classification of Diseases and Related Health Problems. Tabular List, 2022*; Centers for Disease Control and Prevention (CDC), National Center for Health Statistics: Hyattsville, MD, 2022;
